# Delirium and the risk of developing dementia: a cohort study of 12 949 patients

**DOI:** 10.1136/jnnp-2022-328903

**Published:** 2022-05-23

**Authors:** Samuel P Leighton, James W Herron, Eric Jackson, Matthew Sheridan, Fani Deligianni, Jonathan Cavanagh

**Affiliations:** 1 University of Glasgow, Institute of Health and Wellbeing, Glasgow, Glasgow, UK; 2 Imaging Centre of Excellence, Queen Elizabeth University Hospital, NHS Greater Glasgow and Clyde, Glasgow, UK; 3 University of Glasgow, School of Computing Science, Glasgow, UK; 4 University of Glasgow, Institute of Infection Immunity and Inflammation, Glasgow, UK

**Keywords:** DEMENTIA, MEDICINE, MEMORY, NEUROPSYCHIATRY, PSYCHOGERIATRICS

## Abstract

**Background:**

Delirium is an important risk factor for subsequent dementia. However, the field lacks large studies with long-term follow-up of delirium in subjects initially free of dementia to clearly establish clinical trajectories.

**Methods:**

We undertook a retrospective cohort study of all patients over the age of 65 diagnosed with an episode of delirium who were initially dementia free at onset of delirium within National Health Service Greater Glasgow & Clyde between 1996 and 2020 using the Safe Haven database. We estimated the cumulative incidence of dementia accounting for the competing risk of death without a dementia diagnosis. We modelled the effects of age at delirium diagnosis, sex and socioeconomic deprivation on the cause-specific hazard of dementia via cox regression.

**Results:**

12 949 patients with an incident episode of delirium were included and followed up for an average of 741 days. The estimated cumulative incidence of dementia was 31% by 5 years. The estimated cumulative incidence of the competing risk of death without dementia was 49.2% by 5 years. The cause-specific hazard of dementia was increased with higher levels of deprivation and also with advancing age from 65, plateauing and decreasing from age 90. There did not appear to be a relationship with sex.

**Conclusions:**

Our study reinforces the link between delirium and future dementia in a large cohort of patients. It highlights the importance of early recognition of delirium and prevention where possible.

## Introduction

Delirium and dementia are two of the most common causes of cognitive impairment in the elderly population, but their interrelationship is poorly understood.^1^ Dementia is characterised by an irreversible progressive global cognitive decline. It is associated with huge financial and wider societal costs. In the UK, the annual cost of dementia is £35 billion, two-thirds of which is borne by people with dementia and their families.^2^ Delirium is characterised by an acute and fluctuating disturbance in attention and awareness with associated disturbance in cognition (eg, memory deficit, disorientation, language, visuospatial ability or perception), which cannot be explained by another neurocognitive disorder and does not occur in the context of a severely reduced level of arousal, such as coma. It is a serious and life-threatening neuropsychiatric syndrome, which is a direct physiological consequence of another medical condition, substance intoxication or withdrawal, toxins or multiple aetiologies.^3^ Delirium is very common in the elderly and present in up to 50% of patients over the age of 65 admitted to hospital.^4^ Delirium is a clinical diagnosis, which is often under-recognised and frequently overlooked. This has led to a number of high-profile campaigns to increase the awareness and recognition of delirium across the UK and the wider world.^5^

Dementia is the primary risk factor for delirium and delirium is a major risk factor for subsequent dementia.^1 6^ It is not yet clear if delirium is a simply a marker of brain vulnerability, whether the impact of delirium on dementia is derived from its precipitating cause or whether delirium itself leads to permanent neuronal damage. Delirium is preventable in 30%–40% of cases and is, therefore, an important modifiable risk factor for dementia.^4^

Several clinical studies provide evidence to support the relationship between delirium and dementia. A 2010 meta-analysis of two studies (n=241) found that delirium was associated with an increased risk of dementia (RR 5.7, 95% CI 1.3 to 24.0).^7^ A 2021 meta-analysis of six studies (n=901) showed that delirium was associated with increased odds of developing new dementia compared with patients without delirium (OR 11.9, 95% CI 7.29 to 19.6).^8^ The relationship has also been explored in a small population-based cohort study of 553 individuals aged 85+, which found an increased risk of incident dementia following episode of delirium (OR 8.7, 95% CI 2.1 to 35).^9^

However, to date, the field lacks large studies with long-term follow-up of delirium in subjects initially free of dementia to clearly establish outcomes.^1 4^

Our study has two objectives:

1. To estimate the cumulative incidence of dementia among those who experience an episode of delirium but who have not yet been diagnosed with dementia prior to that episode.
2. To model the effect of age at delirium diagnosis, sex and socioeconomic deprivation on the rate of occurrence of dementia among those still at risk (ie, the cause-specific hazard of dementia).

## Methods

We adhere to the Strengthening the Reporting of Observational Studies in Epidemiology (STROBE) and the Reporting of studies Conducted using Observational Routinely-collected health Data (RECORD) statements.^10 11^

We undertook a retrospective cohort study of patients over the age of 65 who had been diagnosed with an index episode of delirium but who had not been diagnosed with dementia prior to their index episode of delirium. Patients from the National Health Service (NHS) Greater Glasgow & Clyde (GG&C) health board were included. Patients with a diagnosis of delirium made before 1 May 2020 were included back as far as the records allowed. The earliest delirium diagnosis was 21 April 1996. Patients were followed from their first episode of delirium up until 1 October 2020 when the data were collected. The primary outcome event of interest was diagnosis of dementia. A competing event, death before dementia diagnosis, was observed. Patients who had not experienced either event before the end of the follow-up period were coded as censored. Patients who experienced their outcome event on the same day as their index delirium diagnosis were considered to have survived 0.5 days.

West of Scotland Safe Haven at NHS GG&C created the study population from the database population. The diagnoses of delirium and dementia were clinical diagnoses based on the International Classification of Diseases 10th Revision made by the treating clinician (see online supplemental materials). Diagnoses could have been made in accident & emergency (A&E), as an inpatient or outpatient or on death. Age at delirium diagnosis, sex and Scottish Index of Multiple Deprivation (SIMD) 2009 quintile (lowest equals most deprived) were included as covariates. SIMD 2009 was based on their most recent postal address. All subjects had information about covariates—there were no missing data. The total number of relevant delirium patients in the NHS GG&C Safe Haven database determined the sample size.

10.1136/jnnp-2022-328903.supp1 Supplementary data

As outlined above, competing risks are present as a participant is at risk of two mutually exclusive events. Using the Kaplan-Meier estimate of the survival function to estimate the incidence function in the presence of competing risks generally results in upward biases in the estimation of the incidence function. Instead, we used the cumulative incidence function (CIF), which allows for the estimation of the incidence of the occurrence of an event (dementia) while taking competing risk (death without a dementia diagnosis) into account. The CIF for the *k*th cause is defined as: CIF*_k_* (*t*)=Pr(*T*≤*t*, *D*=*k*), where *D* denotes the type of event that occurred, and *T* denotes the time from baseline time until the occurrence of the event. The function CIF*_k_* (*t*) denotes the probability of experiencing the *k*th event before time *t* and before the occurrence of a different type of event.^12^

We also modelled the effect of covariates (age at incident delirium, sex and deprivation quintile) on the cause-specific hazard function. The cause-specific hazard function is the instantaneous rate of occurrence of the primary event (dementia) in subjects who have not yet experienced either event (dementia or death without dementia). The exponentiated regression coefficient from the cause-specific hazard model represents the amount of relative change in the cause-specific hazard function associated with a 1-unit change in the covariate. The cause-specific hazard model is well suited to studying the aetiology of a disease.^13^ We fit the cause-specific hazard model by estimating a Cox proportional hazards model and treating subjects who experience a competing event as being censored at the time of occurrence of the competing event. Postmodel assumption testing included testing the proportional hazard’s assumption via Schoenfeld residuals, using the difference in beta values (DFBETAS) to check for influential observations and assessing the functional form of covariates via Martingale residuals. Age had a non-linear functional form, so the final Cox model was refitted using a penalised cubic spline term for age. The results of our postmodel assumption testing are available in online supplemental materials.

10.1136/jnnp-2022-328903.supp2 Supplementary data

All analyses were performed using R, CRAN V.4.0.0^14^ (with the ‘survival’,^15 16^ ‘cmprsk’^17^ and ‘survminer’^18^ packages) and code is available in online supplemental materials.

10.1136/jnnp-2022-328903.supp3 Supplementary data

## Results

**Figure 1:**
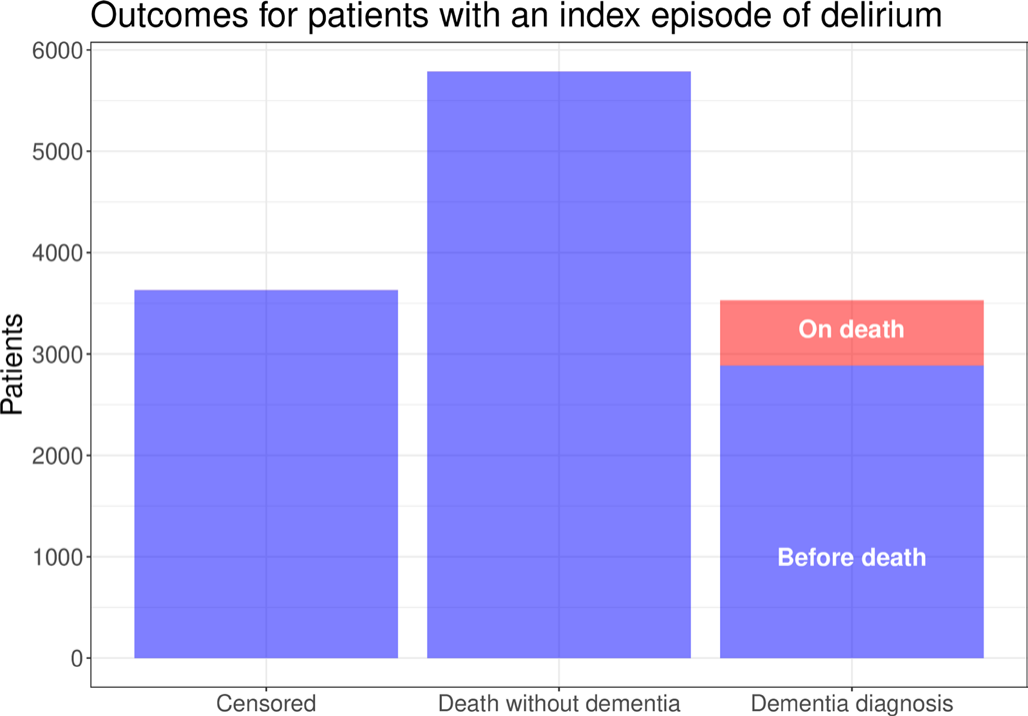
The outcomes for patients with an index episode of delirium follow-up for an average of 741 days (minimum=0.5 days, maximum=8855 days).

12 949 patients with a relevant index episode of delirium followed up for an average of 741 days (minimum=0.5 days, maximum=8855 days) were included in the study. 3530 (27%) of these patients had a subsequent diagnosis of dementia and 5788 (45%) died without a diagnosis of dementia, leaving 3631 (28%) who were coded as censored by the study end date. The diagnosis of dementia was made on death in 643 (18%) of patient who were diagnosed with dementia. This information is summarised in figure 1.

**Figure 2:**
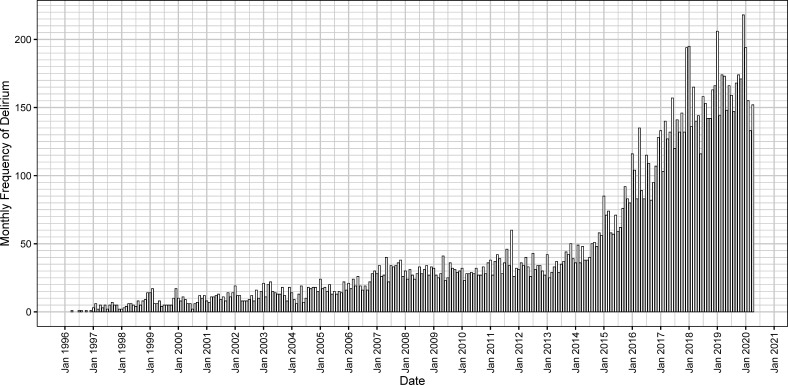
The monthly frequency of new index delirium diagnoses in patients who had not been diagnosed with dementia prior to this episode of delirium.

The diagnosis of new index episodes of delirium increased in frequency over time with some seasonal variation as per figure 2.

Descriptive statistics for the patients in the study are reported in table 1.

**Table 1:** Descriptive statistics for all patients included in the study

Variable	Total sample (n=12 949)	Dementia diagnosis (n=3530)	Death without a dementia diagnosis (n=5788)
Age at index episode of delirium Mean (SD)	82.3 (7.8)	83.2 (7.0)	82.7 (8.1)
Male sex number (%)	5036 (39%)	1262 (36%)	2458 (42%)
SIMD 2009 quintile number (%)	First—4976 (38%) Second—2341 (18%) Third—1986 (15%) Fourth—1673 (13%) Fifth—1973 (15%)	First—1359 (38%) Second—595 (17%) Third—620 (18%) Fourth—515 (15%) Fifth—441 (12%)	First—2247 (39%) Second—1050 (18%) Third—885 (15%) Fourth—672 (12%) Fifth—934 (16%)

SIMD, Scottish Index of Multiple Deprivation.

**Figure 3:**
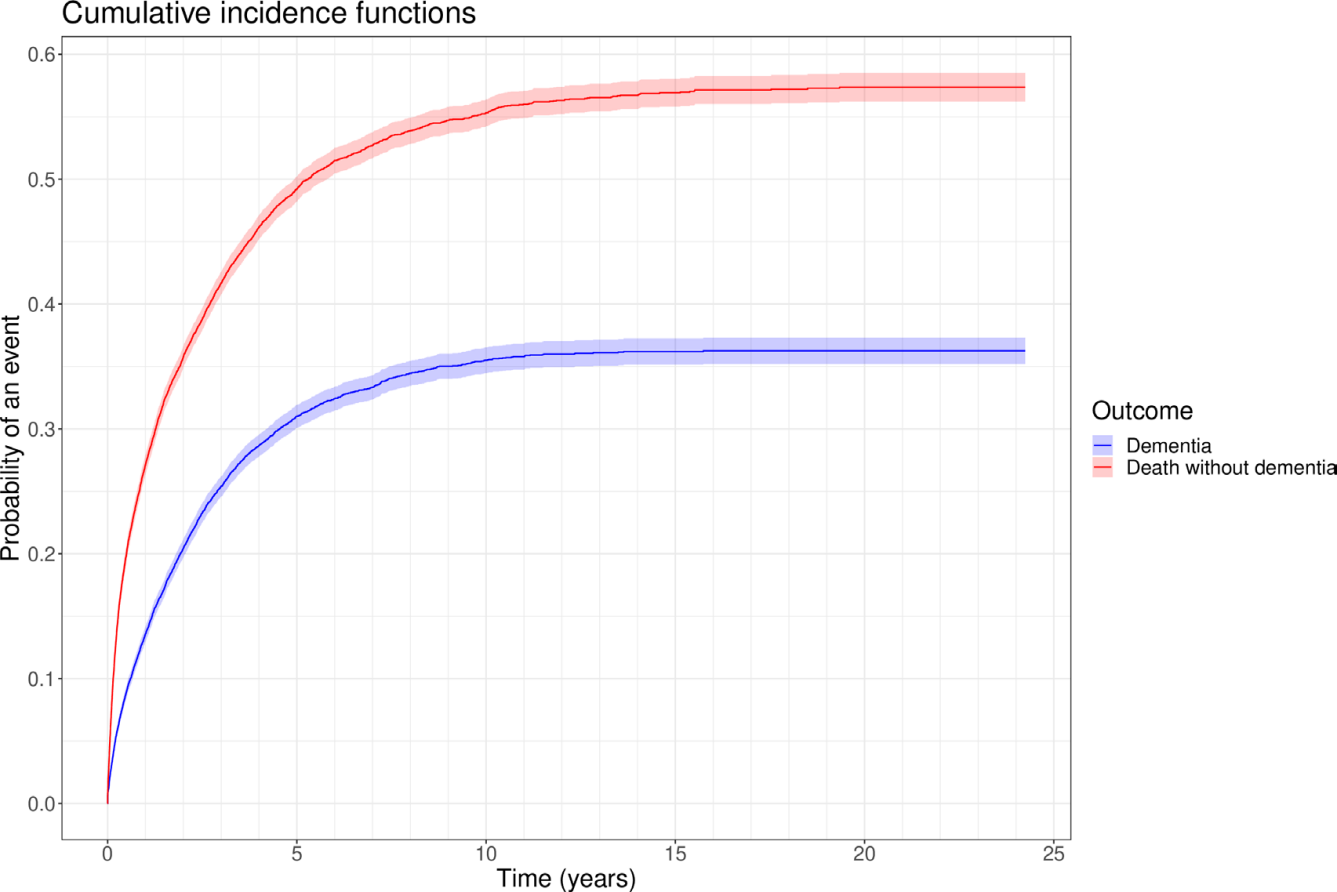
Cumulative incidence function for dementia (blue) and for death without dementia (red) in patients with an index episode of delirium by time in years with 95% CIs.

The estimated cumulative incidences of dementia and for the competing risk of death without a dementia diagnosis are presented in figure 3. The estimated cumulative incidence of dementia, accounting for the competing risk of death without a dementia diagnosis, was 9.0% (95% CI 8.5% to 9.5%) by 6 months, 13.6% (95% CI 13.0% to 14.2%) by a year, 31.0% (95% CI 30.1% to 31.9%) by 5 years, 35.5% (95% CI 34.5% to 36.5%) by 10 years, and 36.3% (95% CI 35.2% to 37.3%) by 20 years. The estimated cumulative incidence of the competing risk of death without a dementia diagnosis was 20.0% (95% CI 19.3% to 20.7%) by 6 months, 27.1% (95% CI 26.3% to 27.9%) by a year, 49.2% (95% CI 48.2% to 50.2%) by 5 years, 55.3% (95% CI 54.3% to 56.4%) by 10 years and 57.4% (95% CI 56.2% to 58.5%) by 20 years.

**Figure 4:**
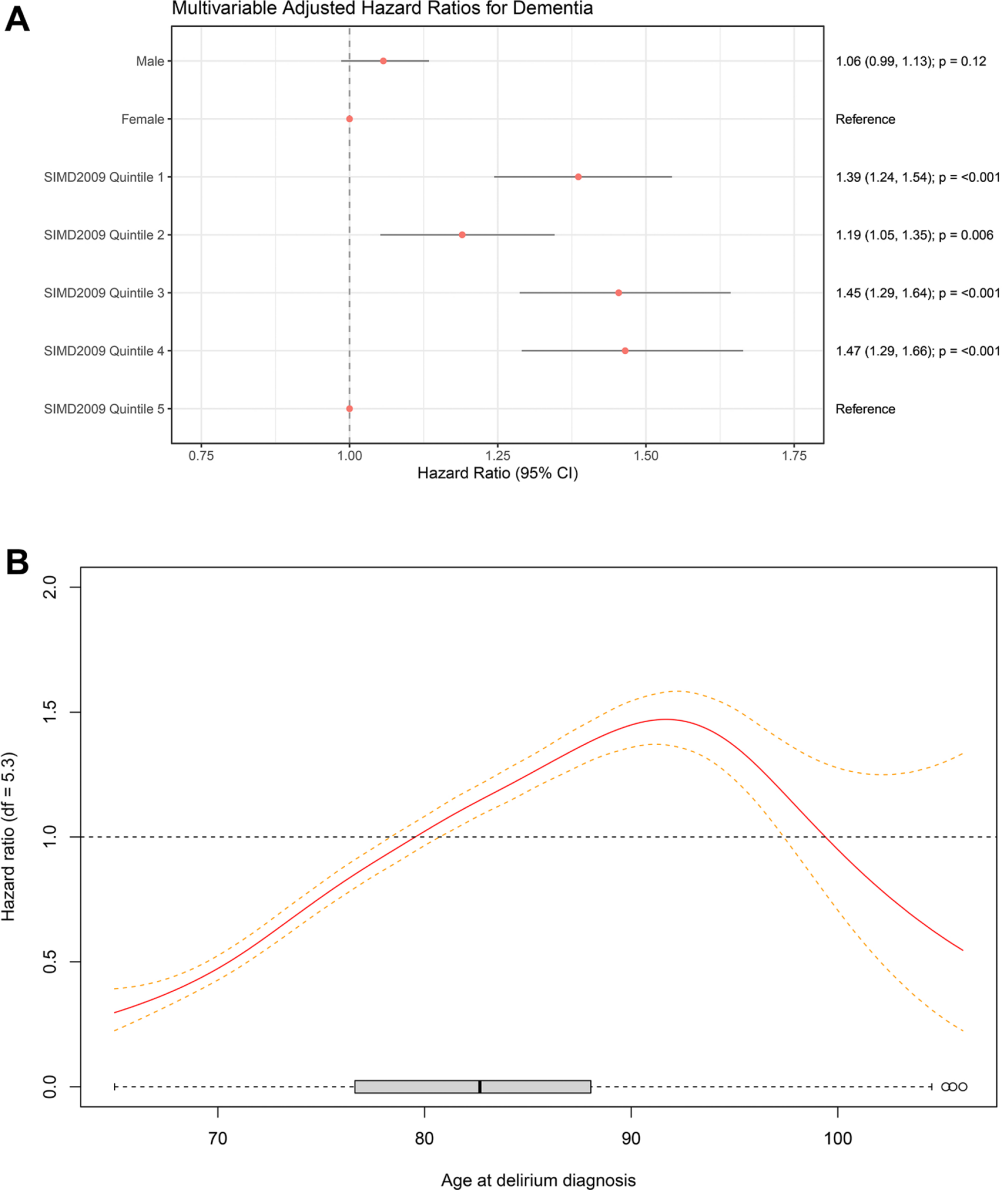
(A) Multivariable adjusted cause-specific hazard ratios for dementia diagnosis in patients with an index episode of delirium. The cause-specific hazard ratios of the four most deprived SIMD 2009 quintiles are greater than the least deprived quintile (reference). There does not appear to be a relationship between sex and cause-specific hazard of dementia in patients with an index episode of delirium. (B) Association of age at delirium diagnosis with cause-specific hazard of dementia in Cox model with penalised spline after multivariable adjustment with 95% confidence intervals (reference 79.5 years; p≤0.001). The cause-specific hazard of dementia increases with age of delirium diagnosis from age 65 until around age 90, when it plateaus then decreases. df, df of freedom; SIMD, Scottish Index of Multiple Deprivation.

The multivariable adjusted cause-specific HRs for sex and SIMD 2009 deprivation quintile are illustrated in figure 4A. The multivariable-adjusted cause-specific HR for age at delirium diagnosis is illustrated in figure 4B.

## Discussion

To the best of our knowledge, this study represents the largest cohort (n=12 949) followed up for the longest period of time (up to 8855 days; mean 741 days) within the published literature examining the new diagnosis of dementia following an episode of delirium. The results show that a first episode of delirium after the age of 65 is associated with a substantial risk of subsequently developing dementia (31% by 5 years). This is in line with data from smaller previously published studies.^7 9 19^ Our data also show that delirium is associated with substantial mortality, in addition to the risk of dementia. This underlines the seriousness of delirium and the importance of prompt diagnosis and treatment of underlying cause. Our research supports the concept of delirium as both an indicator of physiological frailty as well as a possible precipitating and accelerating factor in cognitive and physical decline. Within NHS GG&C, there has been a trend of increases in diagnosis of delirium over time. This may indicate that recent high-profile delirium recognition campaigns are having the desired impact including the Think Delirium campaign, which was introduced in NHS GG&C in 2015.^5 20^ Findings from the Cox-regression analysis show that the multivariable-adjusted cause-specific hazard of dementia among those diagnosed with delirium increases with higher levels of deprivation and also with advancing age, plateauing and decreasing in extremes of age. However, there does not appear to be a relationship with sex.

The most frequent causes of delirium involve significant systemic inflammation. Inflammation is well recognised as a major precipitant of delirium.^21^ There exists an extensive network of mechanisms that allow neuroimmune communication.^22^ In recent years, the effects of inflammatory insult on central nervous structure and function have become increasingly well characterised.^21^ Dementia is a disorder which, except in rare single-gene inherited syndromes, has a complex aetiology involving multiple contributory interacting factors. These include ageing, obesity, diabetes, hypertension and smoking—the common strand to these risk factors is the systemic preponderance of inflammatory molecules.^23^ Inflammation is thought to have a central mechanistic role in the pathogenesis of both Alzheimer’s dementia^24^ and vascular dementia,^25^ the two most common subtypes. While acute inflammation is protective to the brain under most circumstances, prolonged or excess release of proinflammatory molecules within the vulnerable or aged brain may activate various downstream cellular cascades relevant to the emergence of dementia.^26^

These phenomena may be relevant in the context of our findings that support the link between hospitalisation with delirium and subsequent dementia diagnosis. It remains a matter of discussion whether delirium is purely a marker of susceptibility to developing dementia, or unmasks/accelerates unrecognised dementia, or indeed, whether delirium may have direct neurotoxic effects that can be causal in the pathogenesis of dementia.^1^ Evidence from the Vantaa 85+ population-based study may provide evidence to support the latter hypothesis. Neuropathological correlates of dementia such as neurofibrillary tau, β-amyloid plaque burden, vascular lesions, Lewy-body pathology and ApoE4 allele status were not found to be positively associated with subjects who developed dementia following delirium, while in contrast, a strong association existed in those that developed dementia without a delirium history.^9^ Although the Vantaa study was not powered to be conclusive, it may suggest that, in some cases, dementia following delirium represents a different aetiological pathway to the development of dementia, rather than being purely a vulnerability marker/accelerant of pre-existing disease.

In our multivariable analysis, the cause-specific hazard of dementia increases with age of delirium diagnosis from age 65 until around age 90, when it plateaus then decreases. This is consistent with existing evidence in the general population demonstrating a doubling of both the prevalence and instance of dementia every 5–6 years until the age of 90.^27^ Evidence for trends in dementia diagnosis among the oldest old is limited by sample size. However, two large population-based cohort studies found the increases in the incidence of dementia plateau or even decline beyond age 90. It is suggested that among the oldest old, risk factors for dementia may not be related to the ageing process itself but with age-associated risk factors such as hypertension, hyperlipidaemia and heart disease.^28–30^

We found that living in an area of deprivation is associated with an increased cause-specific hazard of developing dementia following an incident episode of delirium after adjusting for age at delirium diagnosis and sex. This supports an earlier finding that the hazard of dementia is increased among those living in areas with higher levels of deprivation in an English population cohort study of 6220 adults over the age of 65.^31^ Unfortunately, we did not have information available to adjust for personal indicators of socioeconomic status like personal wealth, educational attainment or occupation, so we are not able to clearly determine whether individual factors were driving this area deprivation effect. However, previous research has shown that living in an area of higher deprivation is associated with poorer health outcomes even after adjusting for personal wealth, education and employment.^32^

Our study has a number of strengths including the large sample size and long length of follow-up. Furthermore, by virtue of being registry based, our study is pragmatic and the setting is entirely naturalistic. We have properly accounted for the impact of competing risks by using the CIF rather than Kaplan-Meier estimator and we have modelled the effect of covariates on the cause-specific hazard of dementia in those who experience an episode of delirium. We adhere to gold-standard STROBE reporting guidelines.

There were several limitations. The cohort largely consisted of patients diagnosed within secondary care. Only diagnoses made at death were included from primary care. This introduced a selection bias for more severe cases of delirium requiring assessment at A&E, on admission to hospital or on death. Equally, it is possible that our cases could have had earlier incidences of delirium, perhaps within primary care, which were milder and not coded and indexed to our data set. Similarly, the majority of dementia diagnoses were made within secondary care. If patients moved out of NHS GG&C after their index delirium diagnosis but before their outcome occurred, their outcome would not be known except if it was made at death. As such, it is possible that the proportion of patients who developed dementia was underestimated due to attrition bias (patients were censored when they should not have been). Furthermore, in those whose dementia diagnosis was made on death, it is possible that this dementia diagnosis was made in primary care at an earlier time point patient and, thus, dementia survival was overestimated. In addition, our cohort is drawn from all medical records over a specific timeframe rather than being set up as a prospective cohort study. We rely on clinicians accurately and reliably coding the diagnosis of delirium at the point of clinical care being administered rather than trained research assistants. While we believe the system of diagnostic coding to be robust within NHS GG&C, it is likely that some cases of dementia or delirium may be missed or inaccurately diagnosed or coded. For example, there is a clear trend of increasing diagnosis of delirium over time within NHS GG&C. This is unlikely to represent a true increase in the underlying rates of delirium but rather represent an increase in the recognition and coding of delirium, perhaps driven by a number of high-profile delirium recognition campaigns, leading to a general increase in awareness of the condition.^5^ Moreover, our multivariable model lacks several important covariates like medical comorbidities, lifestyle factors like diet and smoking or genetics which have been clearly identified as important risk factors for dementia.^33^ Finally, when we designed our study, we set it up as a cohort study of patients with an incident episode of delirium to determine the risk of dementia, not as a case–control study, with patient with delirium and matched controls without delirium. As such, we were unable to determine the net effect of delirium itself on dementia diagnosis. Future work should consider a case–control design to answer this important question.

In conclusion, our study reinforces the link between delirium and future dementia within a unique and well-powered data set. It has key clinical implications. We have shown that delirium in over 65s carries a 31% risk of developing dementia and an even greater risk of death in the 5-year postdiagnosis. This highlights the importance of recognising delirium and preventing it where possible. Future research is required to determine whether the recognition and early treatment of delirium could reduce the risk of subsequent dementia or death. Moreover, at present, there is no consensus about follow-up and monitoring of cognitive function after an episode of delirium in the elderly. Our findings seem to support closer follow-up of delirium and proactive screening for dementia, but this has implications for service provision, particularly as the population ages. Indeed, it may be that those who experience an episode of delirium represent an ‘at risk’ group who could be candidates for future novel targeted therapies for dementia prevention and early-stage treatment. Finally, important questions about the pathophysiology of delirium remain to be answered. It is unclear whether delirium is a marker or an accelerant of irreversible cognitive decline. The field lacks strong data on the mechanistic relationship between delirium and dementia and indeed the cellular/molecular landscape in delirium and dementia. This is best generated through a combination of neuroimaging approaches, quality animal research and human biomarker studies.^4^

## Data Availability

Data may be obtained from a third party and are not publicly available. The study data is available by application to West of Scotland Safe Haven.
