# Supplementary material for: Delirium and the risk of developing dementia: a cohort study of 12 949 patients
Source: J Neurol Neurosurg Psychiatry. 2022 May 23;93(8):822–7. doi: 10.1136/jnnp-2022-328903 (PMC9304115; doi:10.1136/jnnp-2022-328903)
Supplement: Supplementary data [file jnnp-2022-328903supp001.pdf]

**ICD10 Codes**

Delirium Coding

F05 Delirium not induced by alcohol and other psychoactive substances

Dementia Coding

F00 Dementia in Alzheimer's disease

F01 Vascular Dementia

F02 Dementia in other diseases classified elsewhere

F03 Unspecified dementia

F1073 Residual & Late-onset Psychotic Dementia Due to Use of Alcohol

F1173 Residual & Late-onset Psychotic Dementia Due to Use of Opioids

F1273 Residual & Late-onset Psychotic Dementia Due to Use of Cannabinoids

F1373 Residual & Late-onset Psychotic Dementia Due to Use of Sedatives/Hypnotics

F1473 Residual & Late-onset Psychotic Dementia Due to Use of Cocaine

F1573 Residual & Late-onset Psychotic Dementia Due to Use of Other Stimulants Including Caffeine

F1673 Residual & Late-onset Psychotic Dementia Due to Use of Hallucinogens

F1773 Residual & Late-onset Psychotic Dementia Due to Use of Tobacco

F1873 Residual & Late-onset Psychotic Dementia Due to Use of Volatile Solvents

F1973 Residual & Late-onset Psychotic Dementia Due to Use of Multiple Drugs/Psychoactive Substances
