# Supplementary material for: Delirium and the risk of developing dementia: a cohort study of 12 949 patients
Source: J Neurol Neurosurg Psychiatry. 2022 May 23;93(8):822–7. doi: 10.1136/jnnp-2022-328903 (PMC9304115; doi:10.1136/jnnp-2022-328903)
Supplement: Supplementary data [file jnnp-2022-328903supp002.pdf]

POST-MODEL ASSUMPTION TESTING

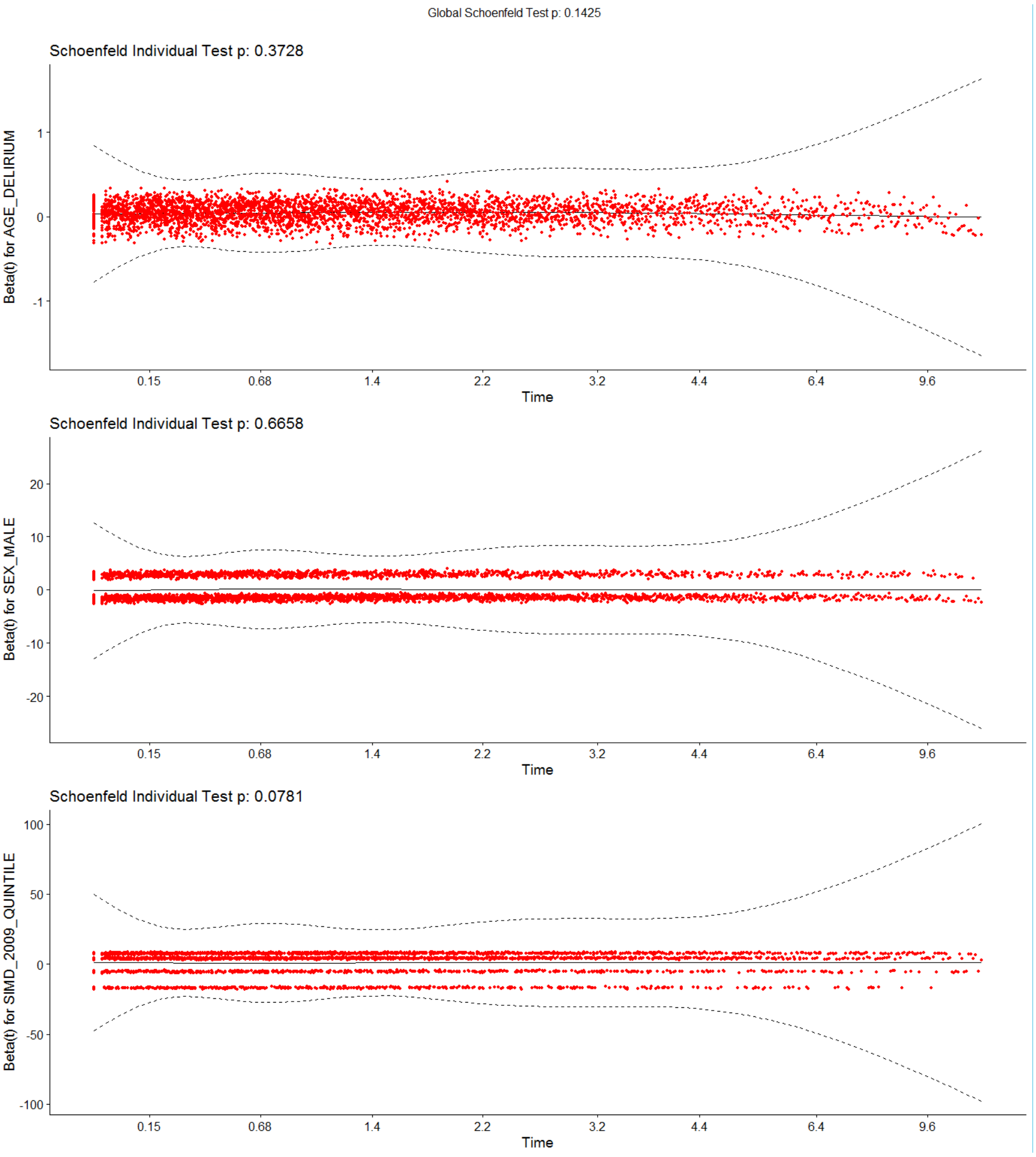

Figure 1 - The proportional hazard assumption is supported by a non-significant relationship between residuals and time for each of the covariates and the global test. The plots of the Schoenfeld residuals are independent of time.

Leighton SP, *et al. J Neurol Neurosurg Psychiatry* 2022; 93:822–827. doi: 10.1136/jnnp-2022-328903

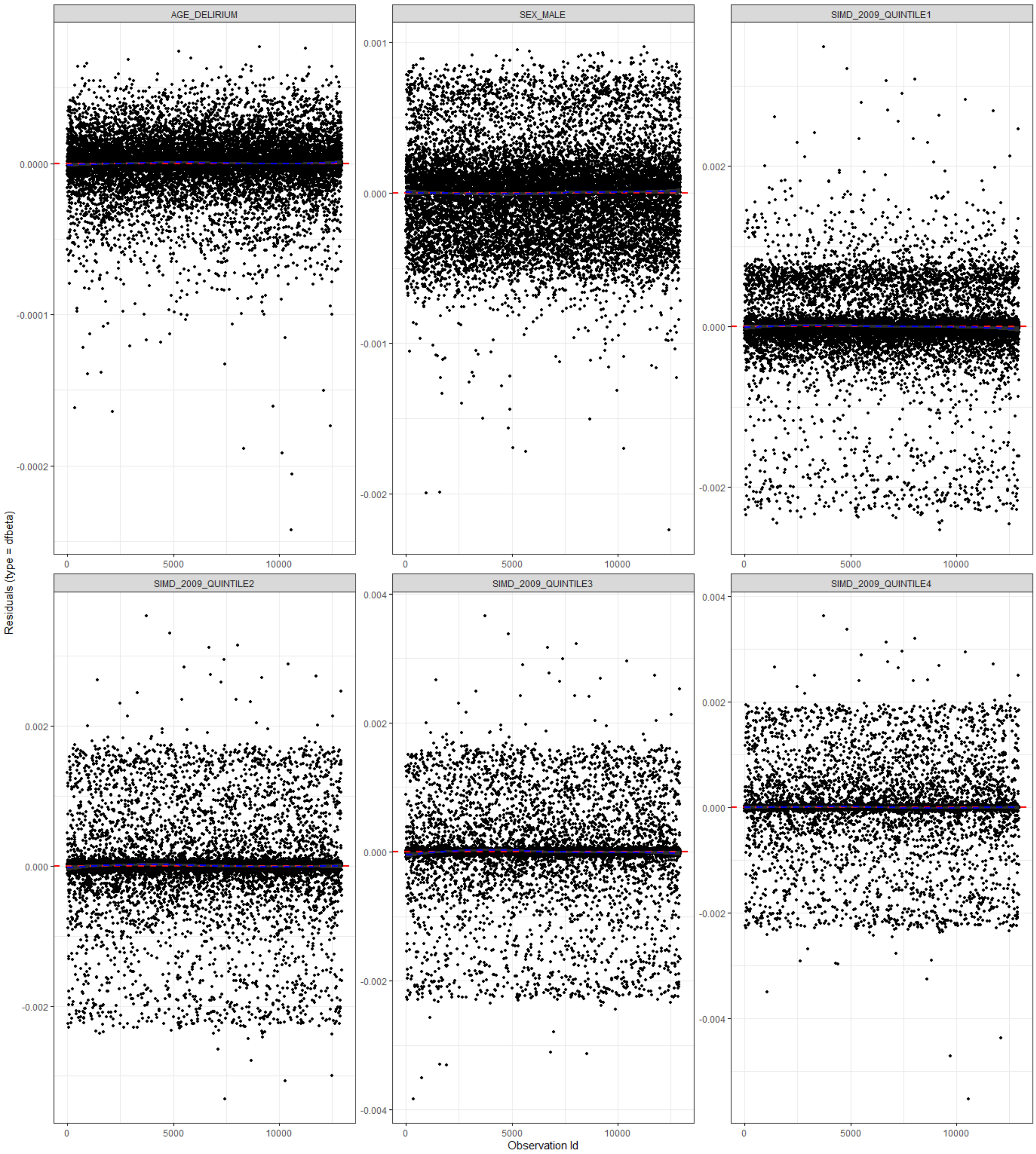

Figure 2 - The above index plots show that comparing the magnitudes of the largest dfbeta values to the regression coefficients suggests that none of the observations are influential individually according to the cut off proposed by Belsley, Kuh and Welch (values larger than 2/sqrt(n) are considered highly influential, in our case values larger than 0.018).[1]

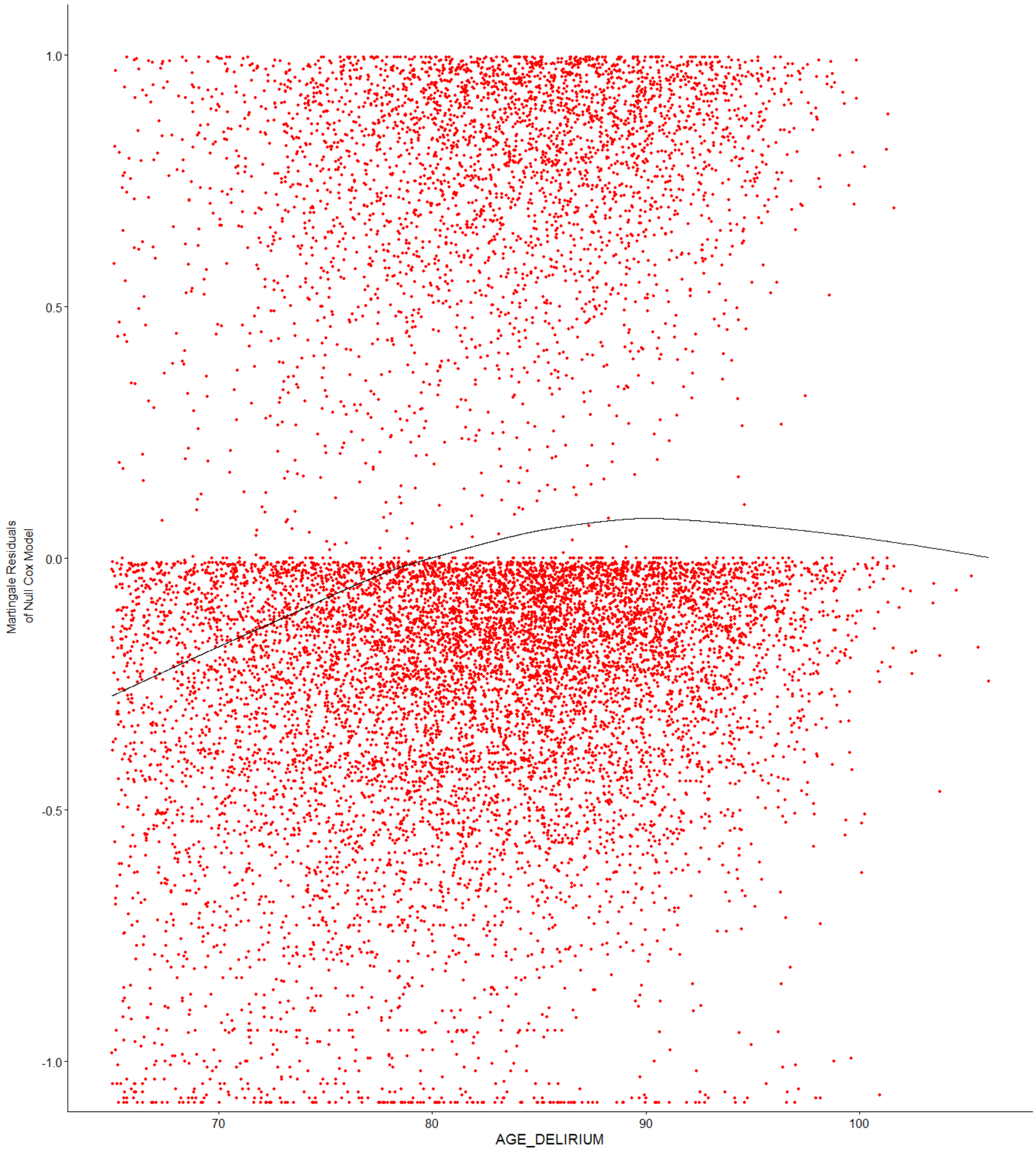

Figure 3 – We assessed for nonlinearity by plotting Martingale residuals of the null cox proportional hazards model against the continuous covariate, age (nonlinearity is not a concern for categorical variables). The fitted line suggests that age has a non-linear functional form, so the final Cox model was refitted using a penalised cubic spline term for age.

References

1 Belsley D a, Kuh E, Welsch RE. *Identifying influential data and sources of collinearity*. New York: : Wiley 1980. doi:10.1002/0471725153
