# Supplementary material for: Delirium and the risk of developing dementia: a cohort study of 12 949 patients
Source: J Neurol Neurosurg Psychiatry. 2022 May 23;93(8):822–7. doi: 10.1136/jnnp-2022-328903 (PMC9304115; doi:10.1136/jnnp-2022-328903)
Supplement: Supplementary data [file jnnp-2022-328903supp003.pdf]

```
library(cmprsk)
library(survminer)
library(readr)
library(ggplot2)
library(scales)
library(dotwhisker)
library(survival)

#working directory
setwd("S:/Sam/Analysis_3/")

options(scipen=999)

data = read_csv("final_ggc_65.csv")
data = data[which(data$TIME_DEMENTIA_DEATH>0),]
data$DELIRIUM_DATE = as.Date(data$DELIRIUM_DATE, format = "%d/%m/%Y")
data$DELIRIUM_YEAR = format(data$DELIRIUM_DATE, "%Y")
data$DEMENTIA_ON_DEATH = as.factor(data$DEMENTIA_ON_DEATH)
data$DEC = data$AGE_DELIRIUM/10
data$SIMD_2009_QUINTILE = as.factor(data$SIMD_2009_QUINTILE)
data$SIMD_2009_QUINTILE = relevel(data$SIMD_2009_QUINTILE, ref = 5)

# TIME_DEMENTIA_DEATH denotes the survival time to the occurrence of the first event
# STATUS_DEMENTIA_DEATH is the event type indicator:
# 1: Dementia diagnosis
# 2: Death before dementia diagnosis
# 0: Censored observation: alive at end of follow-up

# Demographics
summary(as.factor(data$STATUS_DEMENTIA_DEATH))
summary(as.factor(data$DELIRIUM_YEAR))
summary(as.factor(data$SIMD_2009_QUINTILE))
summary(as.factor(data$SEX_MALE))
summary(data$AGE_DELIRIUM)
sd(data$AGE_DELIRIUM)

by(data$SIMD_2009_QUINTILE,as.factor(data$STATUS_DEMENTIA_DEATH), summary)
by(as.factor(data$SEX_MALE),as.factor(data$STATUS_DEMENTIA_DEATH), summary)
by(data$AGE_DELIRIUM,as.factor(data$STATUS_DEMENTIA_DEATH), summary)
by(data$AGE_DELIRIUM,as.factor(data$STATUS_DEMENTIA_DEATH), sd)
by(data$DEMENTIA_ON_DEATH, as.factor(data$STATUS_DEMENTIA_DEATH), summary)

#Stacked bar chart of outcomes
Outcome = c(rep("Censored",2), rep("Dementia diagnosis",2), rep("Death without dementia",2))
dem_on_death = rep(c("No","Dementia diagnosed on death"),3)
Patients = c(3631,0,2887,643,5788,0)
graph_data = data.frame(Outcome, dem_on_death, Patients)
pdf("outcomes.pdf", 10, 7)
ggplot(graph_data, aes(fill=dem_on_death, y=Patients, x=Outcome)) +
  geom_bar(position="stack", stat = "identity", alpha = 0.5) +
```

```
  annotate("text", x=c(3,3), y =c(3250, 1000), label = c("On death","Before death"), color = "white",
size = 6, fontface="bold")+
  theme_bw()+theme(legend.position = "none", text = element_text(size=20), axis.title.x =
element_blank())+
  scale_y_continuous(minor_breaks = seq(0,6000,500), breaks = seq(0,6000,1000))+
  scale_fill_manual(values = c("red","blue"))+
  ggtitle("Outcomes for patients with an index episode of delirium")
dev.off()

#hist(data$DELIRIUM_DATE, breaks = "months", freq = TRUE)
#grid()

#Count by year and month
new = data.frame(table(format(data$DELIRIUM_DATE, "%Y-%m")))
#Append a day
new$Var1 = paste0(new$Var1, "-1")
#TUrn back into a date
new$Var1 = as.Date(new$Var1, format = "%Y-%m-%d")
pdf("delirium.pdf",10,5)
#plot using scale_x_date with 6 month breaks
ggplot(data = new, aes(x=Var1, y=Freq)) + geom_bar(stat="identity", colour ="grey20", fill = "white",
size = 0.25)+
  scale_x_date(labels = date_format("%b %Y"), breaks = date_breaks("12 months"))+theme_bw()+
  theme(axis.text.x = element_text(angle = 90, vjust =0.5, hjust=1))+xlab("Date")+ylab("Monthly
Frequency of Delirium")+
  scale_y_continuous(minor_breaks = seq(0,225,5), breaks = seq(0,225,50))
dev.off()

summary(data$TIME_DEMENTIA_DEATH)

# Figure 1: Plot cumulative incidence functions for dementia and death without dementia
ci_fit = cuminc(ftime = data$TIME_DEMENTIA_DEATH/365.25,
            fstatus = data$STATUS_DEMENTIA_DEATH,
            cencode = 0)
ci_fit

#cumulative incidence 6 months
ci_fit$`1 1`$est[which(ci_fit$`1 1`$time >0.5)][1] #Dementia
ci_fit$`1 1`$est[which(ci_fit$`1 1`$time >0.5)][1]-1.96*sqrt(ci_fit$`1 1`$var[which(ci_fit$`1 1`$time
>0.5)][1])
ci_fit$`1 1`$est[which(ci_fit$`1 1`$time >0.5)][1]+1.96*sqrt(ci_fit$`1 1`$var[which(ci_fit$`1 1`$time
>0.5)][1])
ci_fit$`1 2`$est[which(ci_fit$`1 2`$time >0.5)][1] #Death without dementia
ci_fit$`1 2`$est[which(ci_fit$`1 2`$time >0.5)][1]-1.96*sqrt(ci_fit$`1 2`$var[which(ci_fit$`1 2`$time
>0.5)][1])
ci_fit$`1 2`$est[which(ci_fit$`1 2`$time >0.5)][1]+1.96*sqrt(ci_fit$`1 2`$var[which(ci_fit$`1 2`$time
>0.5)][1])
#cumulative incidence 1 year
ci_fit$`1 1`$est[which(ci_fit$`1 1`$time >1)][1] #Dementia
ci_fit$`1 1`$est[which(ci_fit$`1 1`$time >1)][1]-1.96*sqrt(ci_fit$`1 1`$var[which(ci_fit$`1 1`$time
>1)][1])
```

```
ci_fit$`1 1`$est[which(ci_fit$`1 1`$time >1)][1]+1.96*sqrt(ci_fit$`1 1`$var[which(ci_fit$`1 1`$time >1)][1])
ci_fit$`1 2`$est[which(ci_fit$`1 2`$time >1)][1] #Death without dementia
ci_fit$`1 2`$est[which(ci_fit$`1 2`$time >1)][1]-1.96*sqrt(ci_fit$`1 2`$var[which(ci_fit$`1 2`$time >1)][1])
ci_fit$`1 2`$est[which(ci_fit$`1 2`$time >1)][1]+1.96*sqrt(ci_fit$`1 2`$var[which(ci_fit$`1 2`$time >1)][1])
#cumulative incidence 5 years
ci_fit$`1 1`$est[which(ci_fit$`1 1`$time >5)][1] #Dementia
ci_fit$`1 1`$est[which(ci_fit$`1 1`$time >5)][1]-1.96*sqrt(ci_fit$`1 1`$var[which(ci_fit$`1 1`$time >5)][1])
ci_fit$`1 1`$est[which(ci_fit$`1 1`$time >5)][1]+1.96*sqrt(ci_fit$`1 1`$var[which(ci_fit$`1 1`$time >5)][1])
ci_fit$`1 2`$est[which(ci_fit$`1 2`$time >5)][1] #Death without dementia
ci_fit$`1 2`$est[which(ci_fit$`1 2`$time >5)][1]-1.96*sqrt(ci_fit$`1 2`$var[which(ci_fit$`1 2`$time >5)][1])
ci_fit$`1 2`$est[which(ci_fit$`1 2`$time >5)][1]+1.96*sqrt(ci_fit$`1 2`$var[which(ci_fit$`1 2`$time >5)][1])
#cumulative incidence 10 years
ci_fit$`1 1`$est[which(ci_fit$`1 1`$time >10)][1] #Dementia
ci_fit$`1 1`$est[which(ci_fit$`1 1`$time >10)][1]-1.96*sqrt(ci_fit$`1 1`$var[which(ci_fit$`1 1`$time >10)][1])
ci_fit$`1 1`$est[which(ci_fit$`1 1`$time >10)][1]+1.96*sqrt(ci_fit$`1 1`$var[which(ci_fit$`1 1`$time >10)][1])
ci_fit$`1 2`$est[which(ci_fit$`1 2`$time >10)][1] #Death without dementia
ci_fit$`1 2`$est[which(ci_fit$`1 2`$time >10)][1]-1.96*sqrt(ci_fit$`1 2`$var[which(ci_fit$`1 2`$time >10)][1])
ci_fit$`1 2`$est[which(ci_fit$`1 2`$time >10)][1]+1.96*sqrt(ci_fit$`1 2`$var[which(ci_fit$`1 2`$time >10)][1])
#cumulative incidence 15 years
ci_fit$`1 1`$est[which(ci_fit$`1 1`$time >15)][1] #Dementia
ci_fit$`1 1`$est[which(ci_fit$`1 1`$time >15)][1]-1.96*sqrt(ci_fit$`1 1`$var[which(ci_fit$`1 1`$time >15)][1])
ci_fit$`1 1`$est[which(ci_fit$`1 1`$time >15)][1]+1.96*sqrt(ci_fit$`1 1`$var[which(ci_fit$`1 1`$time >15)][1])
ci_fit$`1 2`$est[which(ci_fit$`1 2`$time >15)][1] #Death without dementia
ci_fit$`1 2`$est[which(ci_fit$`1 2`$time >15)][1]-1.96*sqrt(ci_fit$`1 2`$var[which(ci_fit$`1 2`$time >15)][1])
ci_fit$`1 2`$est[which(ci_fit$`1 2`$time >15)][1]+1.96*sqrt(ci_fit$`1 2`$var[which(ci_fit$`1 2`$time >15)][1])
#cumulative incidence 20 years
ci_fit$`1 1`$est[which(ci_fit$`1 1`$time >20)][1] #Dementia
ci_fit$`1 1`$est[which(ci_fit$`1 1`$time >20)][1]-1.96*sqrt(ci_fit$`1 1`$var[which(ci_fit$`1 1`$time >20)][1])
ci_fit$`1 1`$est[which(ci_fit$`1 1`$time >20)][1]+1.96*sqrt(ci_fit$`1 1`$var[which(ci_fit$`1 1`$time >20)][1])
ci_fit$`1 2`$est[which(ci_fit$`1 2`$time >20)][1] #Death without dementia
ci_fit$`1 2`$est[which(ci_fit$`1 2`$time >20)][1]-1.96*sqrt(ci_fit$`1 2`$var[which(ci_fit$`1 2`$time >20)][1])
ci_fit$`1 2`$est[which(ci_fit$`1 2`$time >20)][1]+1.96*sqrt(ci_fit$`1 2`$var[which(ci_fit$`1 2`$time >20)][1])
```

```
pdf("CIF.pdf", 15, 10)
ggcompetingrisks(ci_fit, multiple_panels = F, conf.int = TRUE) + theme_bw()+
  guides(linetype = FALSE) + scale_y_continuous(minor_breaks = seq(0,1,0.05),breaks = seq(0,1,0.1))+
  scale_x_continuous(minor_breaks = seq(0,25,1), breaks = seq(0,25,5)) +xlab("Time (years)")+
  scale_color_manual(labels=c("Dementia","Death without dementia"), values = c("blue","red"))+
  scale_fill_manual(labels=c("Dementia","Death without dementia"), values = c("blue","red"))+
  theme(text = element_text(size = 20)) + labs(fill = "Outcome", colour ="Outcome")
dev.off()

# confidence intervals are 1.96+/- sqrt variance from cuminc function of cmprsk package as per
Frank Harrell
# discourse.datamethods.org/t/95-ci-around-cumulative-incidence-estimate/3948

#Cause specific hazard model for dementia treats death before dementia as censored
#
#time in years
res.cox.dem = coxph(Surv(TIME_DEMENTIA_DEATH/365.25, STATUS_DEMENTIA) ~ AGE_DELIRIUM +
SEX_MALE + SIMD_2009_QUINTILE, data = data)
summary(res.cox.dem)

#sthda.com/english/wiki/cox-model-assumptions
#test proportional-hazards assumption
test.ph = cox.zph(res.cox.dem)
test.ph
ggcoxzph(test.ph)

#influential observations
ggcoxdiagnostics(res.cox.dem, type = "dfbeta", linear.predictions = FALSE, ggtheme = theme_bw())
#values larger than 2/sqrt(n) are considered highly influential = 0.018

ggcoxdiagnostics(res.cox.dem, type = "deviance", linear.predictions = FALSE, ggtheme =
theme_bw())
#deviance residuals should be roughtly symetrically distributed around zero with a standard
deviation of 1

ggcoxfunctional(Surv(TIME_DEMENTIA_DEATH/365.25, STATUS_DEMENTIA) ~ AGE_DELIRIUM, data
= data, ylim = c(-1,1))
#Is the functional form of age linear. Assess using martingale's residuals from null model

#95%CI for loess fit
smoothSEcurve = function(yy, xx){
  # use after a call to "plot"
  # fit a lowess curve and 95% confidence interval curve
  # make list of x values
  xx.list = min(xx) + ((0:100)/100)*(max(xx) - min(xx))
  # Then fit loess function through the points (xx, yy)
  #  at the listed values
  yy.xx = predict(loess(yy ~ xx), se=T,
              newdata=data.frame(xx=xx.list))
  lines(yy.xx$fit ~ xx.list, lwd=2)
```

```
  lines(yy.xx$fit -
        qt(0.975, yy.xx$df)*yy.xx$se.fit ~ xx.list, lty=2)
  lines(yy.xx$fit +
        qt(0.975, yy.xx$df)*yy.xx$se.fit ~ xx.list, lty=2)
}

#SAME AS ABOVE ggcoxfunctional
res.cox.dem.0 = coxph(Surv(TIME_DEMENTIA_DEATH/365.25, STATUS_DEMENTIA) ~ 1, data = data)
rr.0 = residuals(res.cox.dem.0, type = "martingale")
plot(rr.0 ~ AGE_DELIRIUM, data=data)
smoothSEcurve(rr.0, data$AGE_DELIRIUM)
#Is the functional form of age linear. Assess using martingale's residuals from null model

rr.final = residuals(res.cox.dem, type = "martingale")
plot(rr.final~ AGE_DELIRIUM, data = data)
smoothSEcurve(rr.final, data$AGE_DELIRIUM)
#Is the functional form of age linear. Assess using martingale's residuals from fully adjusted model
including age as covariate

#Age does not have a linear functional form so remodel using a penalised spline term for age
#See https://stats.stackexchange.com/questions/362510 for interpretation of above martingale's
residuals plots

#add spline setting df = 0 then the AIC = (loglik -df) is used to choose an "optimal" degrees of
freedom
res.cox.dem.spline = coxph(Surv(TIME_DEMENTIA_DEATH/365.25, STATUS_DEMENTIA) ~
pspline(AGE_DELIRIUM, df=0) + SEX_MALE + SIMD_2009_QUINTILE, data = data)
summary(res.cox.dem.spline)

#without age to do likelihood ratio test
res.cox.dem.noage = coxph(Surv(TIME_DEMENTIA_DEATH/365.25, STATUS_DEMENTIA) ~ SEX_MALE
+ SIMD_2009_QUINTILE, data = data)
#p value for age term
#according to Robertson
anova(res.cox.dem.spline, res.cox.dem.noage)
#according to stack exchange https://stats.stackexchange.com/questions/197179
anova(res.cox.dem.spline, res.cox.dem)

#Plot the age term on a log-hazard scale
termplot(res.cox.dem.spline, se=T, terms = 1, ylabs = "Log hazard")

pdf("age_spline.pdf",10,7)
#Plot the age term on a hazard scale
predicted = predict(res.cox.dem.spline, type = "terms", se.fit = TRUE, terms = 1)
plot(data$AGE_DELIRIUM, exp(predicted$fit), type ="n", ylim = c(0,2), ylab = "Hazard ratio (df =
5.3)", xlab = "Age at delirium diagnosis")
lines(smooth.spline(data$AGE_DELIRIUM, exp(predicted$fit)), col = "red", lty = 1)
lines(smooth.spline(data$AGE_DELIRIUM, exp(predicted$fit + 1.96 * predicted$se.fit)), col =
"orange", lty = 2)
lines(smooth.spline(data$AGE_DELIRIUM, exp(predicted$fit - 1.96 * predicted$se.fit)), col =
"orange", lty = 2)
```

```
abline(h=1, lty=2)
boxplot(add=T, data$AGE_DELIRIUM, horizontal = T, at = 0, boxwex = 0.1)
dev.off()

#forest plot
#set up data
term = c("Male", "Female", "SIMD2009 Quintile 1", "SIMD2009 Quintile 2", "SIMD2009 Quintile 3",
"SIMD2009 Quintile 4", "SIMD2009 Quintile 5")
estimate = c(1.057, 1, 1.386, 1.190, 1.454, 1.465,1)
conf.low = c(0.9858, 1, 1.2439, 1.0518, 1.2870, 1.2903, 1)
conf.high = c(1.134, 1, 1.544, 1.346, 1.643, 1.664, 1)
plot_data = data.frame(term, estimate, conf.low, conf.high)

pdf("forest.pdf", 10, 5)
#forest plot
dwplot(plot_data, vline = geom_vline(xintercept = 1, colour = "grey60", linetype = 2),
      dot_args = list(color = "#F8766D"), # color for the dot
      whisker_args = list(color = "Grey47") #color for the whisker
      ) +
  ggtitle("Multivariable Adjusted Hazard Ratios for Dementia") +
  theme_bw() +
  xlim(0.75, 1.75) +
  theme(plot.margin = unit(c(0,5,1,1), "cm")) +
  xlab("Hazard Ratio (95% CI)") +
  geom_text(x = 1.82,
          y = 7, label ="1.06 (0.99, 1.13); p = 0.12",
          hjust = 0,
          size = 3) +
  geom_text(x = 1.82,
          y = 6, label ="Reference",
          hjust = 0,
          size = 3) +
  geom_text(x = 1.82,
          y = 5, label ="1.39 (1.24, 1.54); p = <0.001",
          hjust = 0,
          size = 3) +
  geom_text(x = 1.82,
          y = 4, label ="1.19 (1.05, 1.35); p = 0.006",
          hjust = 0,
          size = 3) +
  geom_text(x = 1.82,
          y = 3, label ="1.45 (1.29, 1.64); p = <0.001",
          hjust = 0,
          size = 3) +
  geom_text(x = 1.82,
          y = 2, label ="1.47 (1.29, 1.66); p = <0.001",
          hjust = 0,
          size = 3) +
  geom_text(x = 1.82,
          y = 1, label ="Reference",
          hjust = 0,
```

```
          size = 3) +
  coord_cartesian(clip = "off")
dev.off()
```
